# Feasibility of delivering parent-implemented NDBI interventions in low-resource regions: a pilot randomized controlled study

**DOI:** 10.1186/s11689-021-09410-0

**Published:** 2022-01-05

**Authors:** Sally J. Rogers, Aubyn Stahmer, Meagan Talbott, Gregory Young, Elizabeth Fuller, Melanie Pellecchia, Angela Barber, Elizabeth Griffith

**Affiliations:** 1 Department of Psychiatry Behavioral Sciences, MIND Institute, University California Davis, Davis, USA; 2 Perelman School of Medicine, Center for Mental Health, University of Pennsylvania, Philadelphia, USA; 3 Department of Communicative Disorders, University of Alabama, Tuscaloosa, USA; 4 Department of Developmental Pediatrics, University of Colorado, Anschutz Medical Campus, Boulder, USA

**Keywords:** Early intervention, ASD, Parent-implemented interventions, Parent coaching, Implementation research, ESDM

## Abstract

**Background:**

This implementation feasibility study was conducted to determine whether an evidence-based parent-implemented distance-learning intervention model for young children at high likelihood of having ASD could be implemented at fidelity by Part C community providers and by parents in low-resource communities.

**Methods:**

The study used a community-academic partnership model to adapt an evidence-based intervention tested in the current pilot trial involving randomization by agency in four states and enrollment of 35 coaches and 34 parent-family dyads. After baseline data were gathered, providers in the experimental group received 12–15 h of training while control providers received six webinars on early development. Providers delivered 6 months of intervention with children-families, concluding with data collection. Regression analyses were used to model outcomes of the coach behaviors, the parent fidelity ratings, and child outcomes.

**Results:**

A block design model-building approach was used to test the null model followed by the inclusion of group as a predictor, and finally the inclusion of the planned covariates. Model fit was examined using changes in *R*^2^ and *F*-statistic. As hypothesized, results demonstrated significant gains in (1) experimental provider fidelity of coaching implementation compared to the control group; and (2) experimental parent fidelity of implementation compared to the control group. There were no significant differences between groups on child developmental scores.

**Conclusions:**

Even though the experimental parent group averaged less than 30 min of intervention weekly with providers in the 6 months, both providers and parents demonstrated statistically significant gains on the fidelity of implementation scores with moderate effect sizes compared to control groups. Since child changes in parent-mediated models are dependent upon the parents’ ability to deliver the intervention, and since parent delivery is dependent upon providers who are coaching the parents, these results demonstrated that two of these three links of the chain were positively affected by the experimental implementation model. However, a lack of significant differences in child group gains suggests that further work is needed on this model. Factors to consider include the amount of contact with the provider, the amount of practice children experience, the amount of contact both providers and parents spend on training materials, and motivational strategies for parents, among others.

**Trial registration:**

Registry of Efficacy and Effectiveness Studies: #4360, registered 1xx, October, 2020 – Retrospectively registered, https://sreereg.icpsr.umich.edu/sreereg/

## Background

Specific interventions for young children with or at high likelihood for autism spectrum disorders (ASD) demonstrate powerful effects in reducing intellectual impairment, improving social communication and language development, and improving social skills when initiated in early childhood [10, 11, 13, 30]. However, many of these interventions are not implemented well within community settings due to technical aspects of the models, delivery intensity, and precision of intervention methods. In fact, very few empirically supported models for toddlers with ASD have demonstrated efficacy when assessed via community delivery, and a recent paper reported that children with ASD who receive intervention in community settings have less favorable outcomes than children who receive intervention in clinical/university settings [19]. This might be because the community systems that serve young children often involve low-income and culturally diverse areas and interventions have not been adapted to fit the needs of families in these areas. These characteristics, combined with low funding rates, low service intensity, and staffing difficulties, make it difficult to implement evidence-based practices (EBP) at fidelity.

Part C, the public early intervention system for children under age three in the USA is, by its public and noncategorical nature, the most likely source of early intervention (EI) for the nation’s young children with signs of ASD. The Part C philosophy focuses on having providers support parents to provide intervention for their child during everyday activities. Children with ASD benefit the most from interventions that include parents [13], and parent participation in EI is predictive of long-term outcomes [17]. Parent-implemented interventions lead to positive changes in parent and child outcomes across multiple interventions (e.g., [27]). Such studies, primarily conducted in clinical research settings, have used a multi-stage measurement approach in which (1) provider coaching ability, (2) parent fidelity to the intervention, and (3) child outcomes are all carefully measured. This has led to an understanding that evidence-based coaching models improve parent fidelity to the intervention, and, in turn, child outcomes are linked to parent fidelity to the intervention (e.g., [24, 26, 35, 44]).

However, too seldom do Part C providers use evidence-based parent coaching methods. Part C providers tend to provide direct intervention services to children [6], which allows for little carryover into child daily life and does not realize the intent of Part C services for family learning [2]. The Part C providers in several locations in our study and our community advisory boards told us that they considered young children at high likelihood of ASD to be the most difficult and discouraging children to serve, due to their intense intervention needs, difficulty engaging with the provider, and poor progress (personal communications to SR and AS). Given the significant cost of educating children with ASD [1] and the importance of high-quality intervention at an early age for improving child outcomes, children’s limited access to evidence-based practices (EBP) in Part C EI is a major concern.

Recently, there have been some attempts to move evidence-based, parent-implemented autism interventions into public early intervention systems with some early success [28, 35]. Researchers have partnered with community providers to train them to use parent coaching strategies to teach parents Naturalistic Developmental Behavioral Interventions (NDBI [31];) that fit the context of the community [5]. These interventions show promise for improving social communication outcomes in children with high likelihood of having ASD when delivered by community-based early intervention providers; however, samples are small and more data are needed. In addition, few studies examining parent-implemented interventions have focused on under-resourced communities [34].

For these reasons, we adapted an evidence-based practice in collaboration with community stakeholders study to address the needs of early intervention providers working with parents and their young children with ASD. In order to improve access to evidence-based care, the project targeted low-income sites that were quite distant from the research team, and all interaction was conducted using distance learning technology. To increase feasibility, training of local parent coaches involved self-instruction via internet-based materials and 12–15 h of distance group consultation from a research staff. There was no direct contact between researchers and community providers, parents or children.

The study used a three-phase model, beginning with input from community partners in six sites regarding early intervention processes and needs. Phase Two involved a component analysis of the parent model to determine which of the strategies to emphasize, as well as some pilot work to test the training and coaching intervention methods. Phase Three, the current study, involved a pilot controlled trial involving randomization by agency in four states and enrollment of 35 coaches working in the Part C system and 34 parent-family dyads described here. We worked with research community collaboratives in six states (MT, AZ, CA, CO, PA, AL) to adapt the Early Start Denver Model’s (ESDM) parent coaching strategies for use in low-intensity settings with Part C providers having limited experience with autism. Each state team included a researcher familiar with early intervention and parent coaching, a representative from the state Part C service system, Part C providers and agency administrators, and family members who had participated in Part C with their autistic toddler. These site teams met with the research team regularly through the development stages of the intervention to support our multi-state needs assessment, assist with recruitment and data interpretation and provide feedback on the training modules and HIIYH modules. We used an iterative process to develop our final product for this pilot test that would be generalizable across multiple state systems.

ESDM is one of the very few comprehensive EI models that have been validated and replicated in multiple published, randomized trials (e.g., [8, 24, 26]). A recent meta-analysis of 12 controlled ESDM studies found significant effects of ESDM on cognition and language compared to usual care groups, even though most of the studies involved low-intensity (1 h per week) or group services delivered by parents or professionals [11]. Multiple studies have examined the effects on parents and children of parent-implemented ESDM (P-ESDM) and demonstrated parent fidelity to the techniques and accelerated child learning in language, imitation, and play [38–41].

ESDM was collaboratively adapted for under-resourced Part C communities by a multidisciplinary group of providers, funding agency representatives, parents, and researchers who provided feedback after a review of ESDM manuals and other written materials. Adaptations involved greatly shortening and streamlining training materials and providing them asynchronously via distance learning, simplifying and shortening procedures for developing short-term intervention objectives and progress monitoring tools, and creating video modules that described and illustrated the key strategies for parents to use with children through cartoons and parent-child videos.

Additional adaptations addressed (1) community values (rural Colorado, rural Alabama, rural California, Montana, Arizona, and urban Philadelphia); (2) the limited time Part C providers have for learning, planning, and data collection; (3) the need to reach families with attractive and practical brief audio-visual learning materials that could be accessed through their phones; (4) the need to use a flipped classroom educational approach grounded in the principles of adult learning for flexible provider training; (5) methods for integrating ESDM approaches within the existing Part C Individual Family Support Plan (IFSP) and delivery approaches; and (6) the very limited service intensity delivered in these communities (as low as one 1 h per month). The resulting model was named the Community ESDM, or C-ESDM.

We used an iterative process to develop both the C-ESDM provider training approach and the parent learning materials from three sources: (1) experimental data using component analysis to examine key components of ESDM, (2) survey data from a multi-state, multi-level survey aimed at better understanding Part C services [2], and (3) ongoing discussions with our implementation teams. The component analysis indicated that parents reached fidelity more easily in individual components after learning all the key ESDM components and integrating them into play activities; therefore all four components were included in the training program. The provider training program included methods of measuring child, parent, and provider progress, provider training materials, online parent lessons, and materials. The online materials, “Help is in Your Hands” (HIIYH; www.helpisinyourhands.org), include four modules with 4 lessons per module focused on narrated video examples of families using the strategies during daily routines at home. Four modules cover the following components: (1) Increasing Children’s Attention to People (Positioning; Following the leader; Finding and making attention magnets; Child comfort zone); (2) Increasing Children’s Communication (Talking bodies; Responding to child body language; Gestures and sounds; Following and leading); (3) Creating Joint Activity Routines (Building Joint Activities in four easy steps; Variations on the theme; Joint activity routines without toys); and (4) the ABCs of learning (A = antecedents; B = behavior; C = Consequences). HIIYH includes the core elements of ESDM included in the parent coaching studies which align with the 11 essential common elements shared across NDBIs (Frost, Brian, Gengoux et al., 9). All provider training activities and parent coaching materials were made available online and also covered during providers’ twice-monthly 1-h interactive webinars.

The current study used a small, randomized trial to test the feasibility and promise of the adapted model for use with Part C providers and families in 4 of the 6 participating states (AL; CA; CO; PA). Families had a young child with social communication challenges considered at high likelihood of a future autism diagnosis. The study tested the effects of this low-intensity training approach for Part C providers on three groups: (1) providers’ use of parent coaching strategies, (2) parents’ use of interactive strategies, and (3) toddlers’ developmental skills.

## Methods

The current pilot study examined the effectiveness of C-ESDM delivered in Part C systems across 4 states: Alabama, California, Colorado, and Pennsylvania. Recruitment began at the agency level, with providers nested within agencies, and families recruited from participating providers’ caseloads. Eligible agencies served children 0–3 through their state’s Part C Program. Agencies were recruited via outreach from university partners in each state. Video calls to describe study details were scheduled with all potential providers at an agency. Interested providers were subsequently contacted by study staff and formally screened via phone or video call. Each agency was randomized to either the Community Early Start Denver Model (C-ESDM) or an active comparison group (All About Young Children; AAYC) immediately upon enrollment, using a matched pair, cluster-randomization procedure. After enrolling, providers recruited eligible families from their existing caseload, using a study flyer and recruitment video to provide interested families with study details. Interested families were contacted by the study coordinator via phone and eligible families were consented and enrolled electronically. The current study focuses on evaluating the impact of C-ESDM at 3 levels: providers’ use of parent coaching strategies, parents’ use of interactive strategies, and toddlers’ developmental level.

### Participants

Thirteen agencies, 35 providers (all female), and 34 families enrolled in the project. Seven agencies (14 providers) were randomized to the C-ESDM and six agencies (17 providers) were randomized to the comparison group. Agencies were randomized at the time the agency leader enrolled in the study. Provider and family group assignments were nested within agency assignment. Each state had at least one agency in each condition. The resulting distribution of providers by state and condition was as follows: Comparison: AL = 2, CA = 2, CO = 8, PA = 4; C-ESDM: AL = 4, CA = 6, CO = 3, PA = 6). One agency (with two providers from PA) and an additional provider at a different agency (from AL) withdrew after assignment to the comparison group before providing any demographic or intake data. One provider (C-ESDM from PA) provided intake and demographic data but withdrew before attending any webinars. One provider (comparison group from PA) withdrew after completing demographic information but did not provide an intake session. In all, this left a final sample of 32 providers reporting demographic information and 31 providers with baseline fidelity scores. (See Table 1 for demographic information about providers.) No providers in either the experimental or comparison groups had previously received any ESDM training.

**Table 1:** Provider demographic characteristics, by state and group, shown as percentages of the group

Measure	State				Treatment group	
	AL (*n* = 4)	CA (*n* = 8)	CO (*n* = 11)	PA (*n* = 9)	AAYC (*n* = 14)	CESDM (*n* = 18)
Provider race/ethnicity (%)						
White	50	75	100.0	100	92.9	83.3
Black/African-American	50	–	–	–	–	11.1
Prefer not to answer	–	25	–	–	7.1	5.6
Hispanic or Latino	–	25	–	–	14.3	–
Highest education (%)						
Associate	–	–	9.1	11.1	14.3	–
Bachelor	–	25	9.1	33.3	21.4	16.7
Master	100	62.5	81.8	55.6	64.3	77.8
Doctorate	–	12.5	–	–		5.6
Typical intensity of services provided						
1–2 h per month	100	50	–	–	–	44.4
3–5 h per month	–	50	90.9	100	85.7	55.6
More than 5 h per month	–	–	9.1	–	14.2	–

There are no significant differences between treatment groups on any of these variables

Agency eligibility criteria included (1) agency receives some Part C funding; (2) agency serves low-income families (defined as below the state mean income; (3) agency provides low-intensity services (fewer than 15 h per week); and (4) agency has at least two providers without previous ESDM training willing to participate in the study.

Provider eligibility criteria included (1) employed as an early interventionist at a participating agency; and (2) no previous training in ESDM; serving or will serve one or more children with social-communication delays with high likelihood of ASD. Providers’ formal titles varied, but most were credentialed professionals working as early educators (early childhood special educators, special instructors, or developmental interventionists) or allied health specialists (speech-language pathologists, physical and occupational therapists).

Inclusion criteria for families and children were (1) child chronological age between 12 and 30 months at study intake; (2) child’s provider is concerned about possible ASD and child meets risk criteria on either the Modified Checklist for Autism in Toddlers, Revised (M-CHAT-R [20];) or Infant-Toddler Checklist (ITC [43];); (3) child is ambulatory with unimpaired hand use; (4) child does not have significant motor, medical, vision, or hearing problems or genetic conditions associated with ASD; (5) child receives fewer than 10 hours per week of early intervention (including the EI agency and all other intervention sources such as applied behavior analysis); (6) English is used at least 60% of the time in the home and parent is able to consent and complete questionnaires in English; (7) participating caregiver is child’s legal guardian; (8) participating caregiver is willing to attend scheduled intervention sessions with participating provider; (9) participating caregiver has not previously received ASD-specific parent coaching; and (10) family income reported during initial telephone screen was below the state means as reported on federal website https://aspe.hhs.gov/poverty-guidelines.

### Recruitment

Providers and agency leaders supported recruitment by providing a flyer and a link to a video to potentially eligible families. All agencies had very few children that met eligibility criteria; therefore leaders tried to link potentially eligible children with participating providers as they were referred to the agency. To reduce bias, providers and leaders gave all potentially eligible families information about the study. In response to recruitment challenges, eligibility criteria for children and families were changed midway through the recruitment phase of the study in these ways: (1) allowing increased family income (in the last year we removed all income restrictions), (2) removal of requirement involving (1) provider concerns about ASD risk and (2) removal of the requirement that children meet all ASD risk criteria on a screening tool. Even so, enrolled children did in fact show ASD risk on screeners. Four children were screened using the ITC; all met “concerns” criteria. Twenty-five of the remaining children were screened using the M-CHAT-R and scored with “high” (*n* = 8) or “moderate” (*n* = 6) ASD concerns. The remaining five children were not formally screened following the removal of this requirement as described above. Half of the children were from ethnic or racial minority groups. There were no statistically significant differences between the intervention and comparison groups related to sociodemographics (proportion non-white, maternal education greater than high school, or income of $50,000 or more). Demographic characteristics of all enrolled parents and children in each state are presented in Table 2.

**Table 2:** Child and family participant demographic characteristics, by state and group

Measure	State				Treatment group	
	AL (*n* = 6)	CA (*n* = 8)	CO (*n* = 13)	PA (*n* = 7)	AAYC (*n* = 14)	CESDM (*n* = 20)
Child age at enrollment (*M*, SD)	25.02, 4.58	24.98, 4.42	26.84, 3.77	25.51, 2.85	26.8, 2.35	25.11, 4.52
Child sex (% male)	83.3	37.5	69.2	100	71.4	70.0
Child race/ethnicity (%)						
White	16.7	100	76.9	28.6	64.3	60.0
Black/African-American	83.3	–	15.4	42.9	28.6	30.0
Asian	–	–	–	14.3	–	5.0
Multiple	–	–	7.7	14.3	7.1	5.0
Hispanic or Latino	–	25	23.1	42.9	28.6	20.0
Maternal education (%)						
High school/GED/vocational	16.7	62.5	23.1	57.1	42.9	35.0
Some college	16.7	25	46.2	28.6	28.6	35.0
College degree	16.7	–	30.8	14.3	28.6	10.0
Graduate degree	50	12.5	–	–	–	20.0
Family income (*M*, SD)	43,966; 26,472 Range: 8800–70000	58,875; 31,534 Range: 0–96,000	61,769; 42,528 Range: 20,000–170,000	26,857; 18,685 Range: 8,000–58,000	52,357; 45,771 Range: 0–170,000	49,640; 26,504 Range: 8800–96,000

There are no significant differences between state or treatment groups on any variable

Providers were asked to recommend all potentially eligible families on their caseload, beginning at the time they enrolled in the study and through the end of their training period. Thus, families entered the study at various points in the provider training, whenever an eligible family was added to their caseload and chose to enroll. The 34 enrolled families were spread across 22 individual providers, with the number of enrolled children per provider ranging from 1 to 4. Providers in either group were free to use the materials and methods provided in the training (described below) with any family on their caseload, even if that family did not enroll.

### Procedures

#### Training procedures

##### Intervention group

The C-ESDM intervention learning consisted of five components: (1) providers’ real-time webinars with trained ESDM parent coaches recorded and available for self-study, (2) providers’ group learning through video reviews with the trained coach, (3) parents’ real-time learning during parent coaching with their providers, (4) parents’ independent learning through HIIYH videos and materials including the parent manual [22, 25], which were given to the families and providers, and (5) child learning through interactions with their parents within everyday activities.

Core learning materials:

1. Parent manual “An Early Start for your Child with Autism” [22, 25].
2. Website “Help Is in Your Hands” with its narratives, videos, and exercises (www.helpisinyourhands.org).
3. Provider video materials, training sessions, and tools on the Help Is in Your Hand website.
4. Parent Refrigerator Lists, which cover the main topic of each week’s intervention session.
5. P-ESDM Parent Fidelity of Implementation Scale.
6. P-ESDM Coaching Fidelity of Implementation Scale.
7. Coach’s list of child objectives from the child’s IFSP broken down into 4–5 learning steps.
8. Child session data sheet capturing progress through objectives and learning steps.

C-ESDM providers received access to learning materials, webinars, and video coaching via telehealth that described core coaching techniques and how to apply them to coach parents to implement the C-ESDM strategies. ESDM content knowledge was delivered through the provision of the published parent manuals to all providers and through the HIYYH videos to both providers and parents such that providers did not require content expertise. The providers’ four sessions of group training included methods of measuring child, parent, and provider progress, review of the provider ESDM training materials, and online parent lessons and materials. Prior to beginning training, providers completed brief online knowledge assessments related to the understanding of adult learning principles, early signs of autism, and parent coaching strategies. If they did not receive a score of 80% or better, they reviewed brief videos introducing these concepts prior to beginning training.

Providers initially attended four weekly telehealth group meetings that included a concept presentation, video examples, and discussions with 3-4 other providers from their agency. Meeting leaders were certified ESDM trainers who had developed the C-ESDM procedures and materials. Session topics included: (1) an introduction to HIIYH and Parent Coaching; (2) Parent Coaching structure and strategies; (3) building specific treatment objectives from IFSP goals and simple tracking methods for child progress; and (4) supporting parent learning. ESDM intervention knowledge was gained through a review of the ESDM manual and the HIIYH videos. Additional training content mirrored usual parent coach training with an emphasis on developing measurable goals base on the child’s IFSP and simplification of data and fidelity tracking. Each meeting included both didactic information as well as interactive activities related to the topic. Between-session activities included practice using materials provided (e.g., coding intervention fidelity; practicing with data collection; HIIYH video content) with a family on their caseload.

Once providers completed the first of their initial four weekly content-based webinars, they could begin to use HIIYH with an enrolled family. After the first four webinars, they then met twice monthly for a small group video review with other providers and discussion of the work with their assigned family with their training coach for 2 additional months. These sessions were reduced to once monthly for the final 3 months (6 months of total training). The logic for allowing them to begin with families before they had completed all training was to allow for the providers to practice with their assigned children week by week (and discuss their experiences in the group sessions) as each new concept was taught. The multimodal adult learning approach thus involves didactic learning, video models, direct experiential learning, self-reflection and evaluation, group feedback, and feedback involving the videos from their family sessions. While it would have been ideal for providers to practice with one child and then have data collected from another, there were not enough eligible children in their caseloads or time in their work schedules to allow for this. Enrolled parents received access to the HIIYH parent materials and the ESDM parent manual; providers could use any of the video and written materials and strategies during their sessions with enrolled families. Intervention sessions continued for 6 months for all children on whatever schedule the interventionist and agency had established for the family (this ranged from planned 1 time monthly to 1 time weekly depending on the state and agency).

##### Comparison group

The comparison group received directions to access publicly available online modules (All About Young Children: AAYC, CA Dept Ed, 2013; allaboutyoungchildren.org)of high quality covering early developmental milestones from birth to 60 months in 5 domains: (1) social-emotional development, (2) language development and literacy; (3) number sense; (4) physical development; and (5) approaches to learning. The website included videos with examples of strategies to promote child development that could be viewed by providers and parents. Providers in the comparison group met monthly (for 6 months) via telehealth with a leader (developmental psychology PhD and early childhood specialist) who reviewed the materials covered and provided a structured discussion on each topic but did not offer concrete suggestions for either parent coaching strategies or child interaction strategies. Providers could use the materials in their Part C intervention in any way they wished.

#### Assessment procedures

##### Provider assessments

Providers completed online questionnaires and session videos at study enrollment and exit (6 months later, or whenever their final family completed intervention). The initial, or baseline, provider video taken at enrollment was a session with a consented Part C family who was receiving ongoing intervention with that provider, in order to sample the provider’s current parent-coaching strategies. After training and initiation of intervention with the project children enrolled in experimental or comparison groups, providers recorded each intervention session on a project-supplied iPad and uploaded all the session videos to a secure, HIPAA-compliant website. The final video uploaded by each provider was selected as their “exit”, or post-intervention video. Note that families/children in provider initial videos were not necessarily the same families/children that providers worked with and filmed for the exit videos. Raters naïve to timepoint coded provider fidelity of coaching implementation (FOI) principles from each intake and exit video for both groups of providers. Analysis of provider change in FOI focused on the initial and the final available videos of the provider. The mean number of weeks between the provider initial and final videos was 17.23 weeks (SD 7.03), which did not differ between the groups (*p* > .49). To track the number of hours delivered, providers completed weekly online questionnaires indicating whether a session was scheduled with each family and whether it took place as scheduled.

##### Parent and child assessments

We reached out to our university partners (those participating in the research community partnership that developed C-ESDM) in each state for help with child assessments and recruited seven assessors (all female), including graduate students (*n* = 1) and early intervention professionals (*n* = 6) working in their local communities. These assessors were hired as contractors (not participants) for the project and were naïve to provider group assignment. The study team sent each assessor a kit with a recording device, forms, and necessary toys and stimuli to complete the assessments. The child assessments included two primary components: (1) A parent-child interaction and (2) the assessor-administered ESDM Infant-Toddler Curriculum Checklist (IT-CC [23]; described below). For the parent-child interaction, the assessor asked the parent to play with their child in their typical way. They were asked first to play without any objects, and after that, they were asked to play with their child with a toy either from those at home or from a selection of toys the assessor brought. The parent-child interaction lasted up to 20 min. The assessor then carried out the IT-CC with the child, described in detail below. Each assessment was digitally recorded for later scoring of parent and child behaviors by naïve university coders. In addition to these live interactions, some parent measures were completed online by the parents. For the very few parents who did not complete the online measures, the assessors provided the surveys as paper and pencil measures.

Assessor training procedures included one initial telehealth training with a project member on the assessment procedures. Providers then submitted practice tapes for feedback on administration and scoring until they reached fidelity benchmarks specified for the IT-CC. Following this training, assessors began seeing families. Family contact information was provided to assessors via secure, HIPAA-compliant messaging, and assessors contacted families directly to schedule at a time that was mutually convenient. Assessments were scheduled in families’ homes and lasted approximately 1.5 h. Assessors scored the IT-CC live at the time of assessment administration and submitted copies of their scores and videos of the assessment sessions via a secure website so that their scores could be checked for accuracy by a trained member of the research team. If an item was missing or incomplete, the assessor was contacted directly by the Project Coordinator to clarify.

The entire process of recruiting and training providers and assessors, identifying and enrolling eligible families and children, conducting the intervention, and gathering final data took approximately 1 year. Agencies were enrolled in rolling fashion and all the activities related to conducting the intervention study other than coding and data analysis were completed in a 2-year period.

### Measures

#### Infant-Toddler Checklist (ITC [43];)

The ITC is a 25-item checklist that assesses infants’ language, communication, play skills, and probes for parent concern. Empirically derived cut-offs for concerns range are available for infants 6 through 24 months. The ITC was used as an eligibility screener.

#### Modified Checklist for Autism, Revised (M-CHAT-R, [20])

A 20-item checklist designed to screen for ASD. It provides empirically derived cut-offs for concern and referral recommendations. The M-Chat, including the follow-up interview, was used as an eligibility screener.

#### ESDM Fidelity Checklist [21]

This tool was used to assess parent use of ESDM practices in play with their child. The ESDM Fidelity Checklist consists of 13 items that are each given a score between 1 and 5, with 5 representing more frequent and higher quality use of each ESDM strategy and a total possible range of scores from 12 to 60. The items are (a) management of child attention; (b) ABC teaching format; (c) instructional techniques; (d) Modulating child affect/arousal; (e) management of unwanted behavior; (f) use of turn-taking/dyadic engagement; (g) child motivation is optimized; (h) adult use of positive affect; i. adult sensitivity and responsivity; (j) multiple varied communicative functions; (k) adult language; (l) joint activity and elaboration; and (m) transition between activities (this item was not scored for this study). Trained coders naïve to group and timepoint scored parents on the Fidelity Checklist from the parent-child interaction filmed at the assessments. Coders used this tool to score the play activity without toys and the play activity with toys that the parent carried out during the assessment. A play activity had to last a minimum of 1 min to be coded. Scores were averaged across both activities for an average total parent fidelity of implementation (FOI) rating. Twenty-nine percent of videos were independently coded by both coders for reliability. Intraclass correlation coefficients indicated high reliability: ICC = 0.85 (CI: 0.62–0.95).

#### Coaching Practices Rating Scale (CPRS, [29])

A modified version of the Coaching Practices Rating Scale was used to evaluate provider fidelity of implementation (FOI). Each of the 13 items was rated on a binary scale of present or absent, and these scores were summed for a total of 13 possible points. These behaviors were rated by two coders naive to timepoint and group assignment. Twenty percent of videos were independently coded by both coders for reliability. Intraclass correlation coefficients indicated high reliability: ICC = 0.92 (CI: 0.17–0.98).

#### ESDM Infant-Toddler Curriculum Checklist (IT-CC [23];)

The IT-CC is a criterion-based measure of early development that spans the developmental range from 8 to 30 months and is adapted from the Early Start Denver Model Curriculum Checklist (ESDM [21];). The IT-CC consists of 136 items organized in 9 developmental domains: Gestures Understood, Words Understood, Gestures Produced, Words Produced, Joint Attention, Dyadic Engagement, Imitation, Cognition, and Play Skills. Items are assessed during semi-structured play- and routine-based interactions carried out over approximately 90 min using a standard set of play materials. Each IT-CC item is rated as “acquired”, “partially acquired”, or “unable or unwilling,” based on child behavior during play-based interactions throughout the entire assessment, as well as parent report. On the IT-CC, a score of “acquired” on a given item represents a defined mastery level of that skill and is credited. No other score receives credit. The Cognitive domain was not utilized during the current study after pilot testing indicated the additional required materials were too burdensome for assessors to carry into families’ homes. Thus, final scores for this study consist of one point per ‘acquired’ item, for a total score out of 124 possible points, expressed as a raw score (IT-CC Total Score). A team of gold-standard coders at the primary university site, naive to timepoint and group assignment, scored the IT-CC from videos. (This team was not the same team to code the parent ESDM fidelity videos.)Their scores were used for all analyses, rather than the home assessors’ scores, because of the potential for assessors to become unblinded to family/provider group assignment. Intraclass correlation coefficients of assessors and gold standard coding team indicated high reliability: ICC = 0.93 (CI: 0. 89 to 0.95).

#### Family implementation survey

Upon exit from the study, caregivers completed an implementation survey that asked questions about the feasibility/acceptability of the intervention skills that were taught to them. This survey has been adapted from the literature (Haug, Shopshire, Gruber, & Guydish, 14; Ingersoll & Dvortcsak, 15) and measures the constructs of treatment acceptability, appropriateness, adoption, and feasibility on a Likert scale from 1 = strongly disagree to 5 = strongly agree.

### Analysis

Nine of the 32 families with baseline curriculum scores withdrew sometime after the initial baseline measures and before the final home assessments were completed. Of the three who withdrew in the comparison group, one withdrew due to family stress, one moved out of state, and one was lost to follow-up. Of the 6 who withdrew in the C-ESDM group, two discontinued EI to initiate intensive services, one was moved off the provider’s caseload, one was lost to follow-up, and two were paired with providers who withdrew. This left a final sample of 23 families with outcome data, in comparison to 34 families who provided data from the initial visit which are included in the demographic analyses and descriptions from intake.

A series of regression analyses were used to model outcomes of the provider FOI coaching scores, the parent FOI to ESDM scores, and child total IT-CC scores. Preliminary analyses revealed that there were no site differences on intake variables using all enrolled providers and families/children. (*p* ≥0.06). A nested model-building approach was used to test the null model (accounting only for pretest), followed by the inclusion of group as a predictor to address the primary research question, followed by the inclusion of changes in parent ESDM fidelity, the only planned covariate, and finally the inclusion of any additional covariates. Variables that were significant predictors of outcome were retained in the model. The significance of included variables was examined using changes in *R*^2^ and the *F*-statistic.

The first model tested the impact of the C-ESDM training on the outcome of coaching behaviors. The providers’ initial level of Coaching Practices fidelity and group assignment were included, in that order, to understand the effect of group assignment on provider fidelity of implementation. The second model tested the impact of the C-ESDM intervention on parent ESDM fidelity. The parent’s initial level of ESDM fidelity of implementation and group assignment were included, in that order, to understand the effect of group assignment on parent ESDM fidelity. The third model tested the effect of change in the parent ESDM fidelity on child IT-CC scores. The child’s pretest score, group assignment, and changes in parent ESDM fidelity, the planned covariate, were included in the model in that order, to understand the effect of group assignment and the possible contribution of changes in the changes in parent ESDM fidelity on child outcomes. All interaction terms between pretest variables and group assignment were examined. All statistical analyses were completed using SPSS Statistics V. 26.

## Results

Providers in both conditions attended an average of 78.36% of possible webinars/coaching contacts, which did not differ by group (*t*(29) = 0.86, *p* = 0.93). This translated to a mean of 9.71 h of webinar training/supervision sessions attended (SD = 2.11) by the C-ESDM group and 5.71 h (SD = .47) attended by the comparison group providers. Provider-reported weekly session attendance data indicated no group differences in the number or proportion of family sessions completed (sessions completed: Mean_C-ESDM_ = 11.09, SD_C-ESDM_ = 5.99, Mean_comparison_ = 14.25, SD_comparison_ = 5.68, t(15.72) = 1.71, *p* = 0.26; percent sessions attended: Mean_C-ESDM_ = 54.08%, SD_C-ESDM_ = 20.28, Mean_comparison_ = 64.33, % SD_comparison_ = 12.66, t(16.73) = 1.35, *p* = 0.19).

### Provider FOI outcomes

The first of the regression analyses of coaches’ FOI showed that the initial FOI coaching rating was not significantly related to exit FOI (*β* = 0.24, se = 0.27, *p* = 0.38). To test the hypothesis that inclusion in the C-ESDM intervention would result in higher coaching FOI scores, group was entered into the null model. Participation in the C-ESDM group predicted a significant increase in providers’ coaching FOI compared to the control group (*β* = 4.30, se = 1.40, *p* = 0.007), with a significant improvement in model fit (*R*^2^ change = 0.34, *F* = 9.39, *p* = 0.007). Observed means and standard errors for three primary outcome variables are shown in Table 3.

**Table 3:** Mean (SE) of outcome variables at initial and exit assessments

	C-ESDM		Comparison (AAYC)	
	Pre	Post	Pre	Post
**Number of families**	*n* = 20	*n* = 13	*n* = 12	*n* = 10
**Number of providers**	*n* = 18	*n* = 12	*n* = 13	*n* = 8
Coaching score	4.83 (0.62)	7.67 (0.74)	3.54 (0.93)	3.25 (1.20)
Parent fidelity	3.24 (0.12)	3.66 (0.15)	3.21 (0.12)	3.15 (0.14)
Child IT-CC total score	41.40 (5.45)	59.85 (9.63)	44.75 (7.60)	62.50 (10.27)

ANOVAs showed no significant differences between groups at intake (*p*≥0.24)

### Parent FOI outcomes

The C-ESDM parent group attended on average 11.09 sessions (sd.5.99), which was 54.08% of scheduled sessions (SD 20.28%), and the comparison group attended on average 14.25 (SD = 5.68) sessions, 64.33% (SD = 12.66%) of scheduled sessions. The groups did not differ significantly on number of sessions (*t* = 1.71, df = 15.72, *p* = 0.26) or percentage of scheduled sessions (*t* = 1.35, df = 16.73, *p* = 0.19). The results of this second set of regression analyses showed that pretest parent ESDM FOI was significantly related to posttest FOI (*β* = 0.48, se = 0.22, *p* = 0.04), indicating that parents with higher ESDM FOI scores at the start of intervention (their interaction skills at baseline) also had higher scores at the end of the intervention, and vice versa. To test the hypothesis that inclusion in the C-ESDM intervention would result in higher parent ESDM FOI ratings, group was entered into the null model. Participation in the C-ESDM group predicted a significant increase in parent ESDM FOI compared to the control group (*β* = 0.520, se = 0.20, *p* = 0.02), with a significant improvement in model fit (*R*^2^ change = 0.19, *F* = 6.40, *p* = 0.02).

### Family implementation survey results

Analysis of parent implementation ratings measuring the constructs of treatment acceptability, appropriateness, adoption, and feasibility revealed no differences between groups on the overall ratings: Mean_C-ESDM_ = 3.47, SD_C-ESDM_ = 0.75, Mean_comparison_ = 3.19 SD_comparison_ = 0.86, *t*(15.91) = 0.80, *p* = 0.43) and on any of the scales. Parents rated both the control and C-ESDM interventions as moderately acceptable (*M* = 3.31 and 3.35 respectively), appropriate (*M* = 3.11 and 3.49), feasible (*M* = 3.28 and 3.58), and adoptable (*M* = 3.00 and 3.08 respectively). All subscales had standard deviations around 1.0.

### Child outcomes

The results of the third set of regression analyses indicated that the initial IT-CC total score was significantly related to the exit score (*β* = 1.16, se = 0.14, *p* < 0.01). To test the hypothesis that inclusion in the C-ESDM intervention would result in higher child scores, group was entered into the null model. Participation in the C-ESDM group did not result in a significantly greater change in child scores compared to the comparison group (*β* = 1.17, se = 7.32, *p* = 0.87). Changes in parent ESDM FOI scores across the intervention period, the planned covariate, were not related to child outcomes (*β* = 5.49, se = 6.48, *p* = 0.40). Interactions between initial variable data and group assignment were examined for all analyses; none of these interactions were significant.

## Discussion

### Brief summary

This implementation feasibility study used a research-community partnership approach [5] and was designed and executed in order to answer three questions about an evidence-based parent-implemented distance-learning intervention model for low-income young children with or at high likelihood of having ASD. The questions were (1) could it be learned and implemented at fidelity by community providers after brief group training; (2) could community providers coach parents in ways that effectively transmitted evidence-based skills as measured by the fidelity of implementation measures, in an average of one contact per week or less; and (3) would children of parents receiving the parent coaching model demonstrate positive benefits in comparison to children whose parents received information on child development only. The development of the intervention used a three-phase model, beginning with input from community partners in six sites. Phase two involved a component analysis of the parent model to determine which of the strategies to emphasize and some pilot work to test the training and coaching intervention methods. Phase three involved this pilot-controlled trial involving randomization by agency in four states and enrollment of 35 coaches working in the Part C system and 34 parent-family dyads of whom 50% were from under-represented ethic/racial groups. Families of qualifying children (based on social-communicative delays and ASD risk) were enrolled by their EI providers and initial baseline data on provider coaching, parent-child interactions, and child development were gathered. Providers in the experimental group then received as much as 12–15 h of telehealth training via webinars, group sessions with direct feedback, and asynchronous self-instructional materials, during which they initiated intervention with enrolled families, as well as twice-monthly group video review sessions via telehealth. Comparison group providers received six webinars on various aspects of early development followed by initiation of intervention with children and families.

After approximately 6 months of intervention at whatever schedule the agency typically delivered (ranged from 2 h per week to 1 h per month), video measures of provider interactions with the dyad and videos of parent interactions with child were collected again as was developmental information on children, collected by a naïve evaluator. Results demonstrated significant gains in fidelity to the coaching model of providers in the experimental group compared to those in the control group. Results also demonstrated significant gains in fidelity to the intervention strategies of parents in the experimental groups compared to those in the comparison group, supporting the primary and secondary hypotheses of the study. Gains in provider coaching fidelity were not related to their baseline coaching scores; however, gains in parent intervention fidelity were related to their baseline fidelity scores. There were no significant differences between groups in child developmental scores. Parents in the C-ESDM group did not report the intervention to be less feasible to use, less acceptable, less appropriate, or less adoptable than the community standard intervention, which placed far less responsibility on parents during session than did the experimental intervention.

### Implications

This study focused on adaptation of a well-tested intervention to fit the needs of public agencies, providers, and families in four low-income areas across the country, chosen because these settings have very limited services and the families, many of whom are from under-represented groups, often face many difficulties in accessing high quality intervention for their young children at risk for ASD. The sites involved both urban and rural settings in locations where neither intensive services for young children with ASD, nor expertise in early ASD intervention, were available.

Involvement of community-academic partnerships in various sites allowed for needed guidance of the research team about the needs, strengths, values and priorities of providers and families in each region. Use of distance learning and self-instructional learning activities were  necessary because of: (1) the very limited time allowed by agencies for provider training, (2) the geographic distances involved, and (3) the need to contain costs and develop a method that had sufficient reach to the families and providers in these low-resource areas. These three challenges highlighted several of the novel features of this study, in addition to three more: characteristics involving low-income families in low-income regions, use of telehealth technology for provider training, and lack of any direct contact between the study team and providers or families.

Providers in the C-ESDM group met in small groups with a project coach 1 h every week for the first month of the project, tapering off to once monthly by month six. Community providers delivered all interventions with parents and children; the project coaches never interacted with the family, nor did they provide direct coaching to the providers during sessions. To our knowledge, other parent-mediated implementation studies have not relied on local providers to implement the experimental intervention in low-resource settings, nor have they relied on distance learning and such limited contact to teach the intervention to the coaches. Even though the research project coaches averaged less than 30 min weekly in contact with the provider group over a 6-month period, and no time at all with the parents, both providers and parents in the experimental group demonstrated statistically significant gains with moderate effect sizes compared to the comparison group. Since child changes in parent -mediated models are dependent upon the parents’ ability to deliver the intervention, and since parent delivery is dependent upon providers who are coaching the parents, these results demonstrated that both links of the chain were positively affected by the implementation model being tested here.

However, lack of child change as measured by experimenter-administered measures and the moderate levels of parent use of the intervention outside of sessions suggests that further work is needed on this model. Our group sizes were not large enough to analyze factors influencing child change and the small sample size is a limitation in this study. Factors to consider in future work on this model include amount of contact between parent and provider, amount of practice children experience with parents, amount of parent time spent on learning and practicing between sessions, and motivational strategies for parents. Parents found C-ESDM to be no more complex or challenging to use than the community intervention received by controls, but their ratings of moderate feasibility/usability indicate a need for better support for how to integrate strategies into daily routines. Additionally, providers were gaining comfort with the intervention and parent coaching as they coached parents, and we do not know at what point within the study period the parents reached effective levels of FOI, which may limit the amount of learning opportunities the children are receiving during everyday activities.

Sufficient parent learning time arose as a concern. One of the agencies provided only 1 h per month of contact to children, and if illness, schedules, or holidays required cancelation, no make-up sessions were provided. Given our own, and others’ findings regarding weekly or bi-weekly parent-coaching visits [24, 26, 44], it is difficult to imagine that a parent could learn to embed helpful strategies into natural routines and maintain new learning for a young child with autism symptoms with only 1 h per month of coaching and support. However, the lack of measurable differences in child measures is not an uncommon finding in these kinds of studies. In general, parent-implemented intervention results for young children with autism have been mixed in terms of direct effects on immediate changes in child outcomes [36]. Additionally, many other NDBI community studies have examined therapist-implemented intervention, which has been shown to be more effective across interventions than parent-implementation alone (Nahmias & Mandell, 18). Thus, follow-up research is needed to determine what factors are necessary for changes in parent interaction strategies to permeate child behavioral repertoires in community studies.

A recent replication of these methods, not yet published, in British Columbia, found similar positive results in provider and parent fidelity, as well as significant positive changes in parent questionnaire measures, though not standardized measures, of child progress on multiple measures of development and symptoms in the experimental group. Positive change measured on standardized measures from a parent-mediated intervention is a very high bar. Very few low-intensity parent-mediated models have published direct positive child effects as measured by standardized developmental measures (see [4, 7, 12, 16, 22, 25, 32]). However, since the change in standard scores is widely considered the most rigorous evidence of child improvement, and since many studies of intensive autism intervention have shown that such change is possible, we find it important to continue to strive for this outcome as well.

## Conclusions

The contributions of this study involve (1) methods for reaching providers and parents in distant, low-resource areas, (2) a free public website of learning materials for providers and families, (3) methods that resulted in significant differences in coach and parent behavior related to the intervention strategies, and (4) a low-cost, brief, community training model. Until replication of the C-ESDM model demonstrates positive child-level findings, additional research is needed to further develop and test this approach. However, the primary method in this study—the use of distance technology to transmit strategies successfully from existing efficacious models to community providers and to parents—was both feasible and successful in this study and have been well documented in the literature [3, 33, 37–40, 42]. The use of distance learning methodology to support providers and parents to adopt key features of naturalistic interventions for young children with autism risk can be considered an evidence-based practice.

## Data Availability

The datasets and unpublished materials used and/or analyzed in this study are available from the corresponding author on reasonable request.

## Abbreviations

NDBI: Naturalistic Developmental-Behavioral Intervention
ASD: Autism spectrum disorder
EI: Early intervention
EBP: Evidence-based practice
ESDM: Early Start Denver Model
P-ESDM: Parent-implemented Early Start Denver Model
IFSP: Individualized Family Service Plan
C-ESDM: Community Early Start Denver Model
HIIYH: Help Is In Your Hands
AAYC: All About Young Children
M-CHAT-R: Modified Checklist for Autism in Toddlers, Revised
ITC: Infant-Toddler Checklist
IT-CC: Infant-Toddler Curriculum Checklist
HIPAA: Health Insurance Portability Accountability Act
CPRS: Coaching Practices Rating Scale
